# Which exercise strategies improve postpartum depression? A systematic review and meta-analysis of randomized controlled trials

**DOI:** 10.3389/fpubh.2026.1876038

**Published:** 2026-08-27

**Authors:** Jun-Jun Qiao, Jing-Tong Zhang, Zheng Zhang, Jin-Peng Wang, Xin Liu

**Affiliations:** 1 Department of Sports Science, Ningxia Normal University, Guyuan, China; 2 Department of Physical Education, Kyonggi University, Suwon, Republic of Korea; 3 Department of Sports Science, Yancheng Institute of Technology, Yancheng, China; 4 Department of Social Organization Management, Secretariat of Jiangsu Sports Federation, Nanjing, China; 5 Yangzhou Children's Amateur Sports School, Yangzhou, Jiangsu Province, China

**Keywords:** exercise, meta-analysis, mind–body exercise, postpartum depression, randomized controlled trials

## Abstract

**Objective:**

Postpartum depression adversely affects both mothers and infants and remains an important issue in maternal health. This meta-analysis examined the overall effects of exercise interventions on postpartum depressive symptoms and explored whether programme characteristics influenced treatment outcomes.

**Methods:**

A systematic search of major databases was conducted to identify randomized controlled trials evaluating exercise interventions for postpartum depression. Sixteen studies met the inclusion criteria. Pooled effects were calculated as standardized mean differences (SMDs) with 95% confidence intervals (CIs) using meta-analytic models. Subgroup analyses were performed according to intervention format, exercise modality, initiation timing, weekly frequency, intervention duration, and reporting of delivery mode. Publication bias was assessed using funnel plots and Egger's regression test.

**Results:**

Sixteen randomized controlled trials were included in the analysis. Overall, exercise interventions were associated with a significant reduction in postpartum depressive symptoms (SMD = −0.69, 95% CI −1.02 to −0.36). In subgroup analyses, mind–body exercise showed the largest effect, while aerobic exercise was also associated with symptom improvement. Interventions lasting 12 weeks or less showed stronger effects than longer programmes. No statistically significant subgroup differences were observed for intervention format, weekly frequency, timing of initiation, or reporting of delivery mode, although studies initiating exercise postpartum and those involving vaginal delivery tended to show greater benefit. Sensitivity analyses indicated that the pooled findings were stable.

**Conclusion:**

Exercise interventions may help reduce postpartum depressive symptoms. The findings suggest that exercise modality and programme duration may influence effectiveness, with interventions lasting no more than 12 weeks showing greater benefit. These results support the inclusion of structured exercise in postpartum care while highlighting the need for further high-quality trials to determine optimal exercise prescriptions for postpartum women.

**Systematic review registration:**

PROSPERO, identifier: CRD420251160668.

## 1 Background

Low mood, loss of interest, and cognitive difficulties are common manifestations of depression (1), a prevalent mental disorder that can markedly impair everyday functioning. The Global Burden of Disease study identifies depression as a major cause of disability worldwide, with consequences that extend beyond individual health to family wellbeing and social productivity (2). Although pharmacological and psychological treatments remain the main approaches to care, access to these services is often limited by concerns related to availability, acceptability, tolerability, and safety (3).

Postpartum depression is a major concern in perinatal mental health. It commonly occurs within the weeks or months following childbirth and may persist during the first postpartum year, with reported prevalence ranging from about 10–20% in different populations (4–6). Its effects are not confined to maternal emotional health, but may also influence mother–infant interaction, breastfeeding, and parenting practices. Children of mothers with postpartum depression may be more likely to experience later cognitive, emotional, and behavioral difficulties (7). At the same time, many postpartum women face barriers to treatment, including concerns about medication use during lactation, insufficient access to psychological care, and broader sociocultural constraints (8).

Exercise has been proposed as a practical non-pharmacological approach for reducing depressive symptoms (9, 10). Previous research suggests that physical activity may contribute to symptom improvement through multiple pathways, including effects on neuroplasticity, hypothalamic–pituitary–adrenal axis regulation, sleep, fatigue, and self-efficacy (11). For postpartum women, exercise may also promote physical recovery after childbirth, provide opportunities for social engagement, and support adjustment to the maternal role. As a result, exercise-based interventions have been increasingly examined in relation to postpartum depression (12).

Despite growing interest in this area, the available studies differ considerably in exercise modality, timing of initiation, training frequency, and programme duration. This variation makes it difficult to determine which intervention characteristics are most closely associated with better outcomes. Therefore, this study aimed to systematically evaluate the effects of exercise interventions on postpartum depressive symptoms and to explore whether specific programme characteristics modify treatment effects. Clarifying these issues may help inform more targeted exercise recommendations for postpartum women.

## 2 Meta-analysis data

This meta-analysis was performed according to the Preferred Reporting Items for Systematic Reviews and Meta-Analysis statement and the Cochrane Collaboration Handbook. The protocol was registered on PROSPERO (CRD420251160668).

### 2.1 Data sources and searches

A systematic literature search was performed in the Cochrane Library, Embase, PubMed, and Web of Science from database inception to October 2025. The search process was conducted independently by two reviewers (ZJT and QJJ), and any discrepancies were resolved through discussion; when needed, a third reviewer (ZZ) was consulted. The search strategy combined Medical Subject Headings (MeSH), where applicable, with free-text terms related to the postpartum period, depressive symptoms, and exercise or physical activity. The full search strategies for each database are presented in Supplementary Material 1.

### 2.2 Inclusion and exclusion

Study eligibility was determined according to the PICOS framework. We included studies involving pregnant or postpartum women that examined any exercise- or physical activity-based intervention and compared it with a non-exercise control condition, including usual care, standard health education, wait-list control, or no intervention. Eligible studies were required to report depressive symptoms using a validated psychometric instrument. Only full-text, peer-reviewed randomized controlled trials (RCTs) were considered.

Studies were excluded if they involved populations other than pregnant or postpartum women, lacked an eligible control group, assessed multicomponent or non-exercise interventions for which the independent effect of exercise could not be isolated, did not provide sufficient intervention information or extractable outcome data, or did not evaluate depressive symptoms. Protocols, trial registrations, unpublished studies, studies including participants with major comorbidities likely to interfere with depressive symptom assessment, and articles not published in English were also excluded.

### 2.3 Assessment of risks of bias

The risk of bias of the included studies was assessed independently by two reviewers (ZJT and QJJ) using the Cochrane Collaboration tool. The following domains were evaluated: random sequence generation, allocation concealment, blinding of participants and personnel, completeness of outcome data, selective reporting, and other potential sources of bias. Any disagreements were resolved through discussion, with consultation from a third reviewer (ZZ) when necessary. The detailed results of the risk-of-bias assessment are presented in Supplementary Material 2.

### 2.4 Data extraction

Using a standardized data extraction form, two reviewers (ZJT and QJJ) independently extracted all relevant information from each included study to ensure accuracy and consistency. The use of a uniform template minimized extraction errors and enhanced the reliability of the dataset. Discrepancies were resolved through discussion, with a third reviewer (ZZ) consulted when consensus could not be reached. This rigorous and systematic approach ensured the collection of comprehensive and dependable data, forming a robust empirical basis for the subsequent meta-analysis.

### 2.5 Assessment of overall effect size

Meta-analytic calculations were performed in Review Manager (RevMan, version 5.3). As depressive symptoms were measured using different validated scales across studies, pooled effects were expressed as standardized mean differences (SMDs) with 95% confidence intervals (CIs). Hedges' g was used to estimate effect sizes, with negative values representing greater improvement in depressive symptoms following exercise intervention. Effect sizes were calculated using the Practical Meta-Analysis Effect Size Calculator. Statistical heterogeneity was evaluated using the Q test and the *I*^2^ statistic; *I*^2^ values of 25%, 50%, and 75% were interpreted as low, moderate, and high heterogeneity, respectively. Depending on the level of heterogeneity, either a fixed-effect or random-effects model was applied. Statistical significance was set at *p* < 0.05.

### 2.6 Subgroup analysis of exercise intervention programmes

Six predefined subgroup analyses were conducted to examine whether the effects of exercise interventions on postpartum depressive symptoms differed according to programme characteristics. Studies were grouped by organizational format (individual vs. group-based), exercise modality (aerobic, combined aerobic–strength, mind–body, or monitoring-based activity), timing of intervention initiation (prenatal vs. postnatal), weekly exercise frequency (<3 sessions, 3 sessions, or >3 sessions), total intervention duration (<12 weeks, 12 weeks, or >12 weeks), and delivery mode (natural birth vs. not explicitly reported). For each subgroup, pooled effect sizes were calculated as Hedges' g with 95% confidence intervals using Review Manager (RevMan, version 5.3). Statistical significance was defined as *p* < 0.05, and heterogeneity was assessed within each subgroup.

## 3 Result

### 3.1 Search process

A total of 2,061 records were identified through systematic searches of electronic databases. After removing 97 duplicates using reference management software, 1,964 records remained for title and abstract screening. During this phase, 1,823 records were excluded according to predefined eligibility criteria, including 1,711 articles with clearly irrelevant titles and 112 existing meta-analyses or systematic reviews.

**Figure 1:**
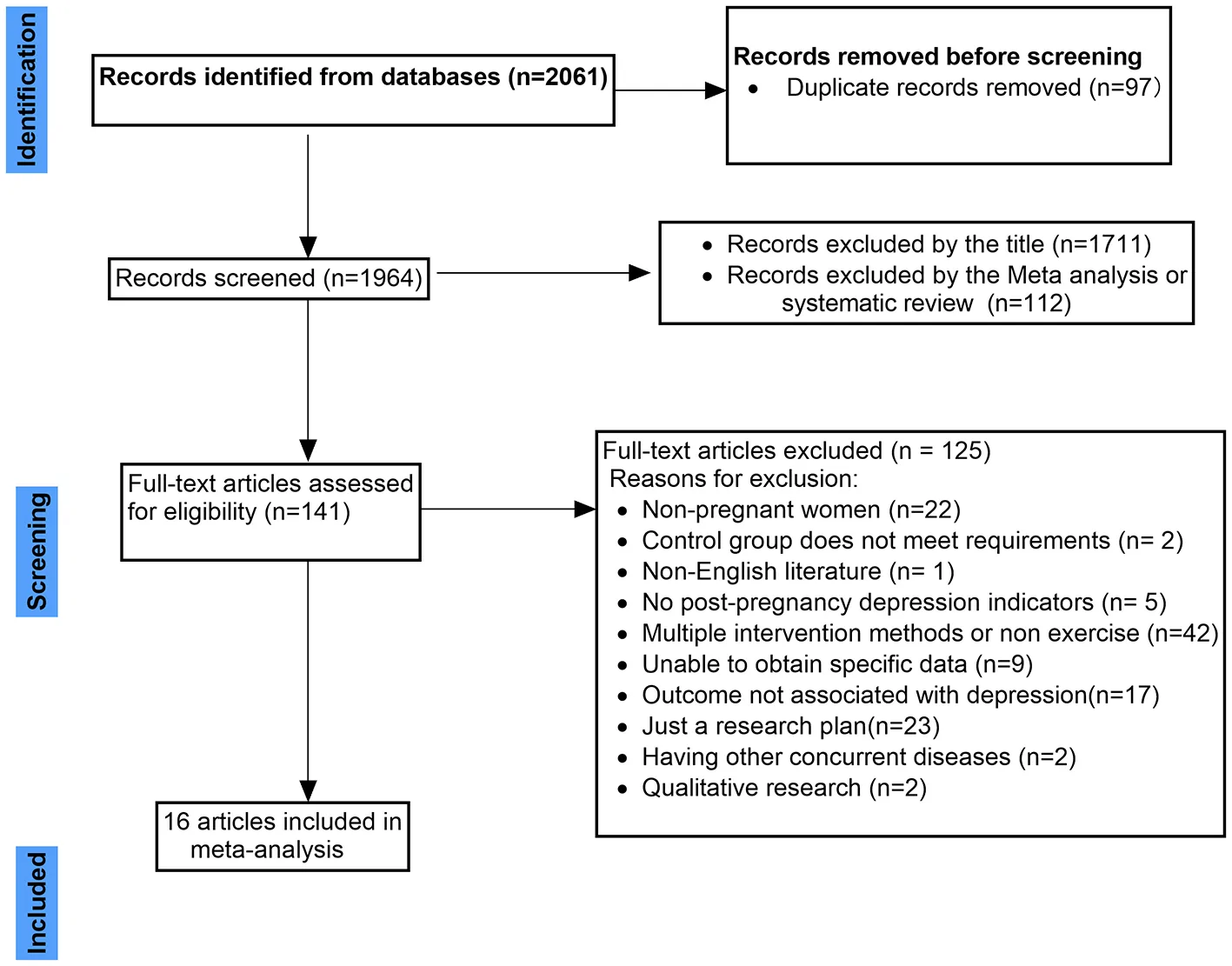
Flowchart and selection of studies.

The full texts of the remaining 141 articles were retrieved for detailed eligibility assessment. Following this review, 125 articles were excluded for the following reasons: non-pregnant or non-postpartum populations (*n* = 22); inappropriate control groups (*n* = 2); non-English publications (*n* = 1); absence of postpartum depression–related outcome measures (*n* = 5); multicomponent or non-exercise interventions in which the independent effect of exercise could not be determined (*n* = 42); insufficient or non-extractable data (*n* = 9); outcomes unrelated to depression (*n* = 17); studies limited to research protocols or registrations (*n* = 23); serious comorbidities likely to confound depression assessment (*n* = 2); and qualitative study designs (*n* = 2). Ultimately, 16 randomized controlled trials met all inclusion criteria and were incorporated into the systematic review and meta-analysis. The full study selection process is presented in Figure 1.

### 3.2 Characteristics of the included studies and participants

The characteristics of the 16 studies included in this meta-analysis are summarized in Table 1. Detailed information is provided on sample size, mean participant age, intervention and control group design, type of exercise intervention, intervention duration and frequency, as well as the depression outcome measures used in each study.

**Table 1:** Characteristics of the included studies and participants.

Studies	Sample size (IG/CG)	Age range (IG/CG)	IG type	Frequency/duration	Outcome measures
Armstrong, K 2004	9:10		Pram walking	3 times weekly/12 weeks	EPDS
Badon, S. E 2025	50:49	31.9 ± 5.1/32.7 ± 4.5	eHealth PA intervention	3 months	PHQ-8
Walczak-Kozłowska, T 2025	34:19	31.35 ± 4.25/32.26 ± 4.21	HIIT	3 times weekly/8 weeks	BDI
Uçakci Asalioglu, C 2024	25:27	29.48 ± 3.65/26.22 ± 3.78	Progressive muscle relaxation exercise	3 times weekly	EPDS
Teychenne, M 2021	32:30	33.6 ± 3.7/33.0 ± 3.7	Aerobic exercise	12 weeks	EPDS
Saeedi, S 2023	20:20		Aerobic exercise	3 times weekly/12 weeks	EPDS
Ramsey, M 2024	30:36		eHealth PA combined yoga–strength–aerobic exercise	6 months	PHQ-8
Özkan, S. A 2020	34:31	28.90 ± 4.83	Combined strength–aerobic exercise	more than 5 times weekly/4 weeks	EPDS
Mohammadi, F 2015	36:36		Stretching exercise	3 times weekly	EPDS
Lewis, B. A 2024	56:55	28.02 ± 5.60/31.44 ± 5.31	Fitbit monitors exercise	more than 5 times weekly/12 weeks	EPDS
Kim, H. B 2022	8:8	39.71 ± 2.01/38.14 ± 1.39	Pilates	2 times weekly/8 weeks	EPDS
Heh, S. S 2008	33:30		Stretching exercises	3 times weekly/8 months	EPDS
Daley, A. J 2015	43:42	31.7 ± 5.3/29.3 ± 5.7	Aerobic exercise	6 months	EPDS
Daley, A 2018	146:133	27.5 ± 6.3	Aerobic exercise	1 times weekly	EPDS
Coll, C. V. N 2019	192:387	27.2 ± 5.5/27.3 ±5.5	Combined strength–aerobic exercise	3 times weekly/16 weeks	EPDS
Forsyth, J 2017	11:11	25.0 ± 5.1/27.0 ± 5.5	Combined strength–aerobic exercise	1 times weekly/12 weeks	EPDS

IG, Intervention Group; CG, Control Group; BDI, Beck Depression Inventory; PHQ-8, Patient Health Questionnaire-8; EPDS, Edinburgh Postnatal Depression Scale; HIIT, High-intensity interval training.

### 3.3 Risks of bias

Of the sixteen included studies, thirteen were judged to have a low risk of bias in random sequence generation, whereas three were rated as unclear due to insufficient methodological reporting. Allocation concealment was assessed as low risk in eleven studies and unclear in five. Blinding of participants was uniformly rated as high risk, reflecting the inherent challenges of blinding in exercise-based behavioral interventions. Outcome assessor blinding was evaluated as low risk in six studies, unclear in six, and high risk in four. Fifteen studies demonstrated a low risk of incomplete outcome data, with one study assessed as unclear. Selective reporting was judged as low risk in thirteen studies and unclear in three. For nine studies, the risk of other potential biases remained unclear owing to inadequate information. Comprehensive assessments for each domain of bias are presented in Supplementary Material 2.

### 3.4 Meta-analysis

#### 3.4.1 Baseline period test

The randomized controlled trials included in this review employed different validated instruments to assess depressive symptoms, including the Edinburgh Postnatal Depression Scale (EPDS), the Beck Depression Inventory (BDI), and the Patient Health Questionnaire-8 (PHQ-8). Owing to the variation in measurement scales and scoring ranges across these tools, direct comparison of mean scores between studies was not feasible. To ensure comparability, baseline depressive symptom levels were synthesized using the standardized mean difference (SMD).

**Figure 2:**
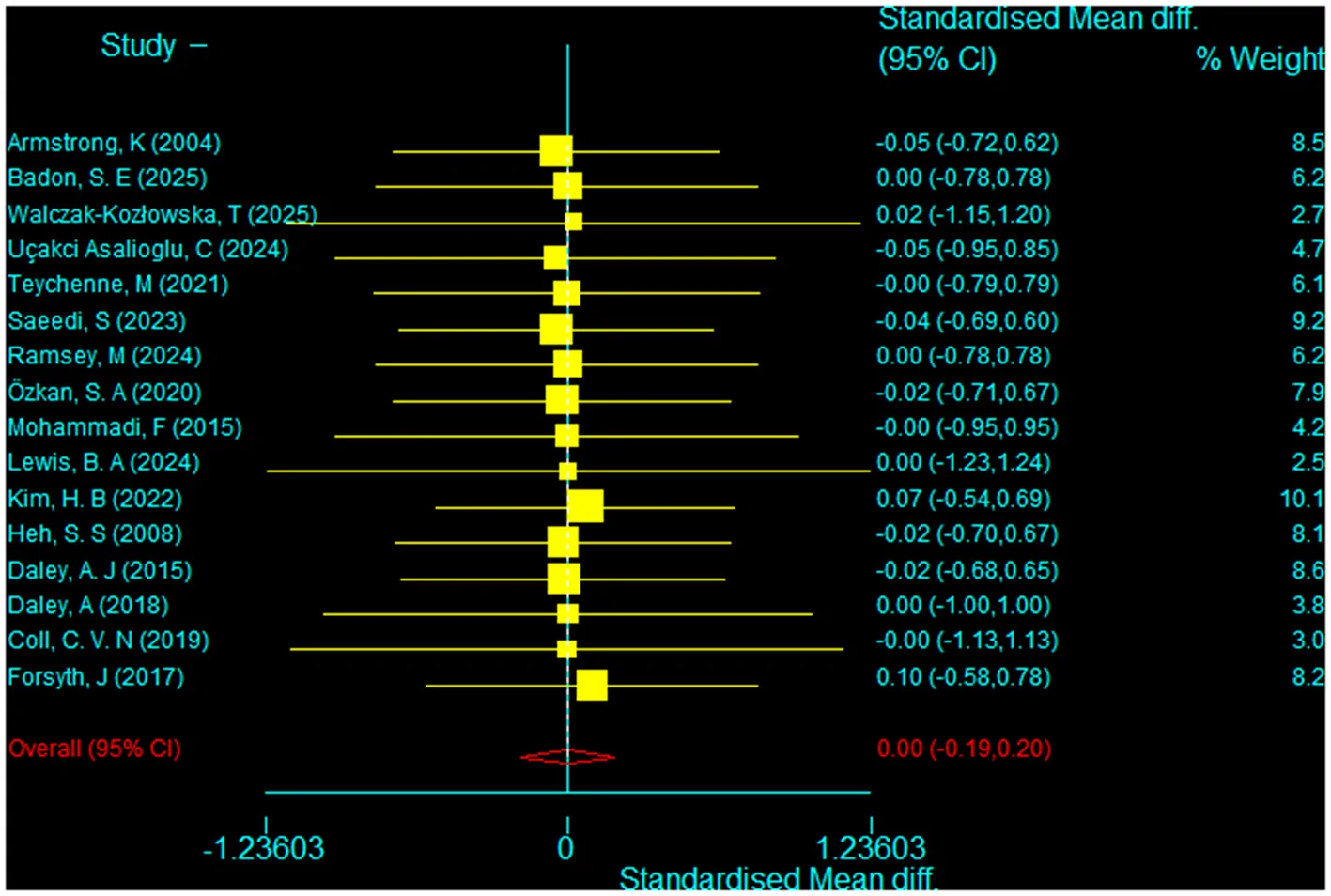
Baseline period test.

The baseline forest plot showed that depressive symptoms were broadly comparable across studies prior to intervention. Most SMD estimates were close to zero, and their 95% confidence intervals crossed zero, indicating no significant differences between experimental and control groups at study entry. The pooled effect size was SMD = 0.001 (95% CI: −0.193 to 0.196), and the statistical test confirmed the absence of baseline differences (*z* = 0.01, *p* = 0.992). Heterogeneity was negligible (χ^2^ = 0.20, *p* = 1.000; Tau^2^ = 0), suggesting that baseline depressive symptom levels were consistent across the included trials. Details are presented in Figure 2.

#### 3.4.2 Meta-analysis result

**Figure 3:**
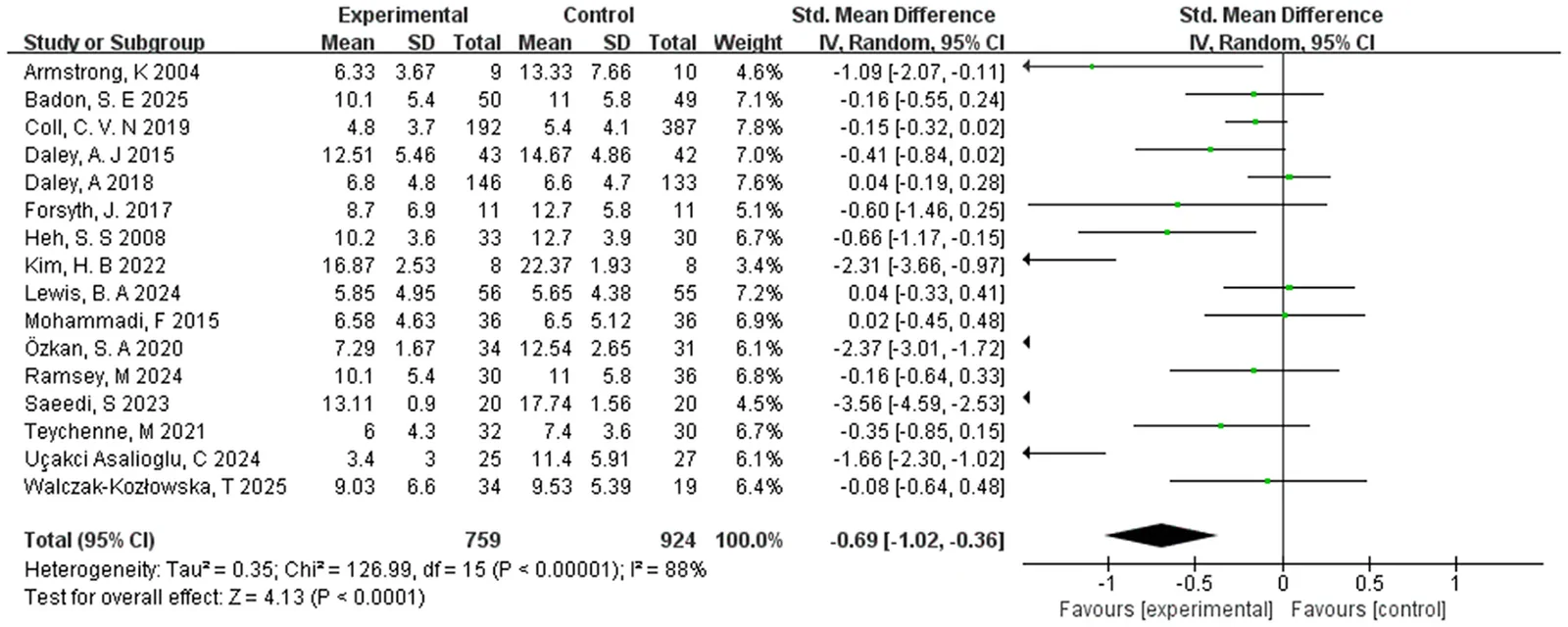
Forest plot of Exercise for Postpartum Depression, reflecting considerable variation in effect sizes across studies.

Analysis of 16 randomized controlled trials demonstrated that exercise interventions exerted a significant beneficial effect on postpartum depressive symptoms. The pooled effect size was SMD = −0.69 (95% CI: −1.02 to −0.36), with strong statistical significance (*Z* = 4.13, *p* < 0.0001), indicating that exercise programmes effectively reduced postnatal depressive symptoms compared with control conditions. However, substantial heterogeneity was observed (χ^2^ = 126.99, df = 15, *p* < 0.00001; *I*^2^ = 88%), as shown in Figure 3.

This heterogeneity is likely attributable to differences in intervention characteristics—such as exercise modality (aerobic, combined aerobic–strength, or mind–body), training dose (frequency and duration), timing of intervention initiation (prenatal vs. postnatal), participant characteristics (e.g., mode of delivery and recovery status), and delivery format (individual, group-based, or digital). These methodological and population-level differences may contribute to the observed variability in outcomes.

To evaluate the robustness of the pooled effect, a sensitivity analysis was performed by sequentially excluding individual trials. The overall effect size remained stable, and heterogeneity metrics showed no substantive changes, indicating that the findings were not disproportionately driven by any single study. This stability supports the reliability of the primary conclusions regarding the efficacy of exercise interventions in alleviating postpartum depressive symptoms.

#### 3.4.3 Publication bias test

**Figure 4:**
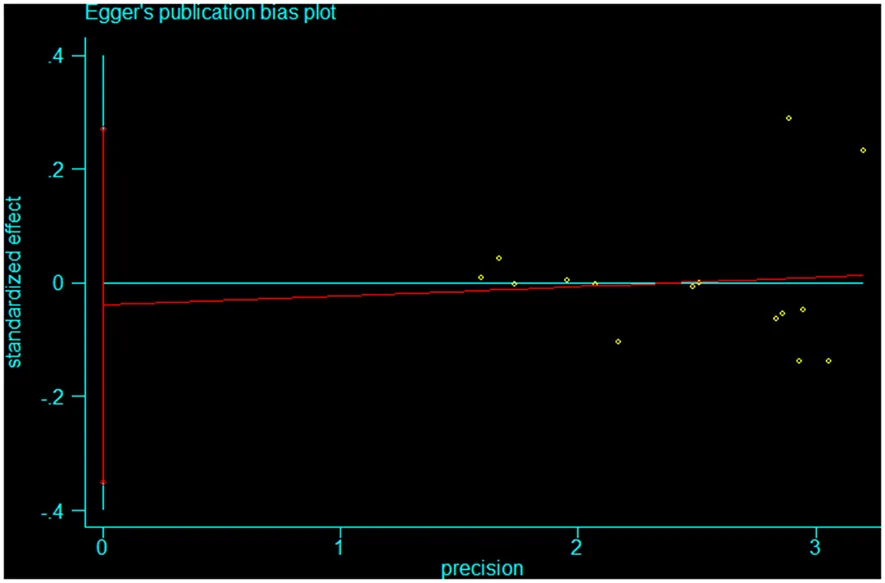
Egger's publication bias plot.

As shown in Figure 4, a funnel plot was constructed to provide an initial assessment of publication bias. The distribution of study estimates appeared broadly symmetrical, with no marked asymmetry, suggesting a low likelihood of publication bias. Given the relatively small number of included trials, visual inspection alone may be insufficient; therefore, the Egger regression test, a method suited to small-sample meta-analyses, was performed to further evaluate potential bias.

**Figure 5:**
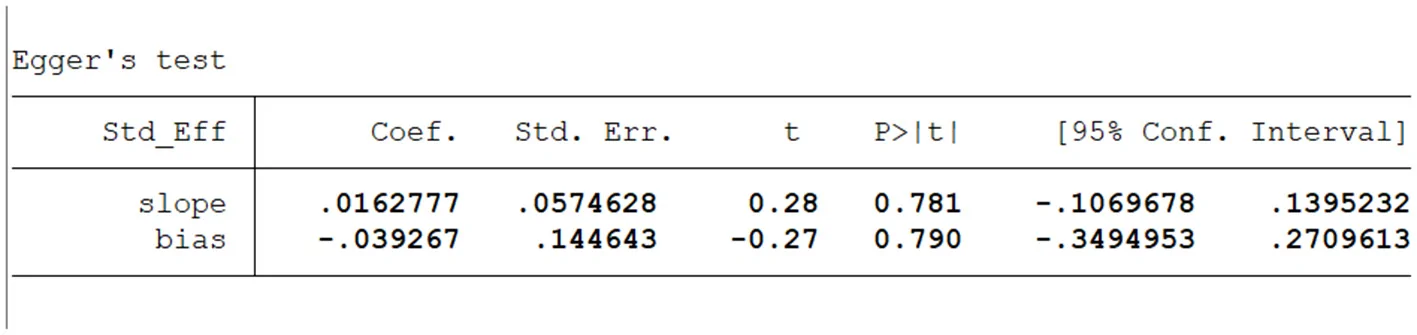
Egger's test.

The Egger test results are presented in Figure 5. The bias coefficient was −0.039 (*t* = −0.27, *p* = 0.790), indicating no statistically significant small-study effects or publication bias. Similarly, the slope coefficient (*p* = 0.781) provided no evidence of systematic deviation. The 95% confidence interval for the bias term (−0.349 to 0.271) crossed zero, further supporting the absence of publication bias. Taken together, the visual symmetry of the funnel plot and the non-significant Egger test results indicate that publication bias is unlikely in this meta-analysis, reinforcing the robustness of the overall findings.

#### 3.4.4 Subgroup analysis

The study sought to examine whether specific characteristics of exercise interventions influence their effectiveness in alleviating postnatal depression. To address this question, six predefined subgroup analyses were conducted: (1) intervention format, comparing individualized versus group-based delivery; (2) exercise modality, including aerobic exercise, combined aerobic–strength training, mind–body interventions, and monitoring-based physical activity; (3) timing of intervention initiation, distinguishing programmes commencing during pregnancy from those initiated postpartum; (4) training frequency, categorized as fewer than three sessions per week, three sessions per week, or more than three sessions per week; (5) intervention duration, grouped as less than 12 weeks, 12 weeks, or more than 12 weeks; and (6) reporting of delivery mode, incorporating studies that specified vaginal delivery and those that did not report delivery mode. These subgroup analyses were designed to systematically investigate whether variations in intervention design or participant characteristics contribute to differences in the observed effects of exercise on postnatal depressive symptoms.

##### 3.4.4.1 Intervention format

The study first compared the effectiveness of different intervention formats in reducing postnatal depressive symptoms, specifically contrasting group-based and individual exercise interventions. Both formats demonstrated significant beneficial effects. The pooled effect size for group-based interventions was SMD = −0.82 (95% CI: −1.50 to −0.14, *p* = 0.02), while individual interventions yielded an effect size of SMD = −0.69 (95% CI: −1.15 to −0.23, *p* = 0.003). These findings indicate that both approaches are effective in mitigating postnatal depression.

**Figure 6:**
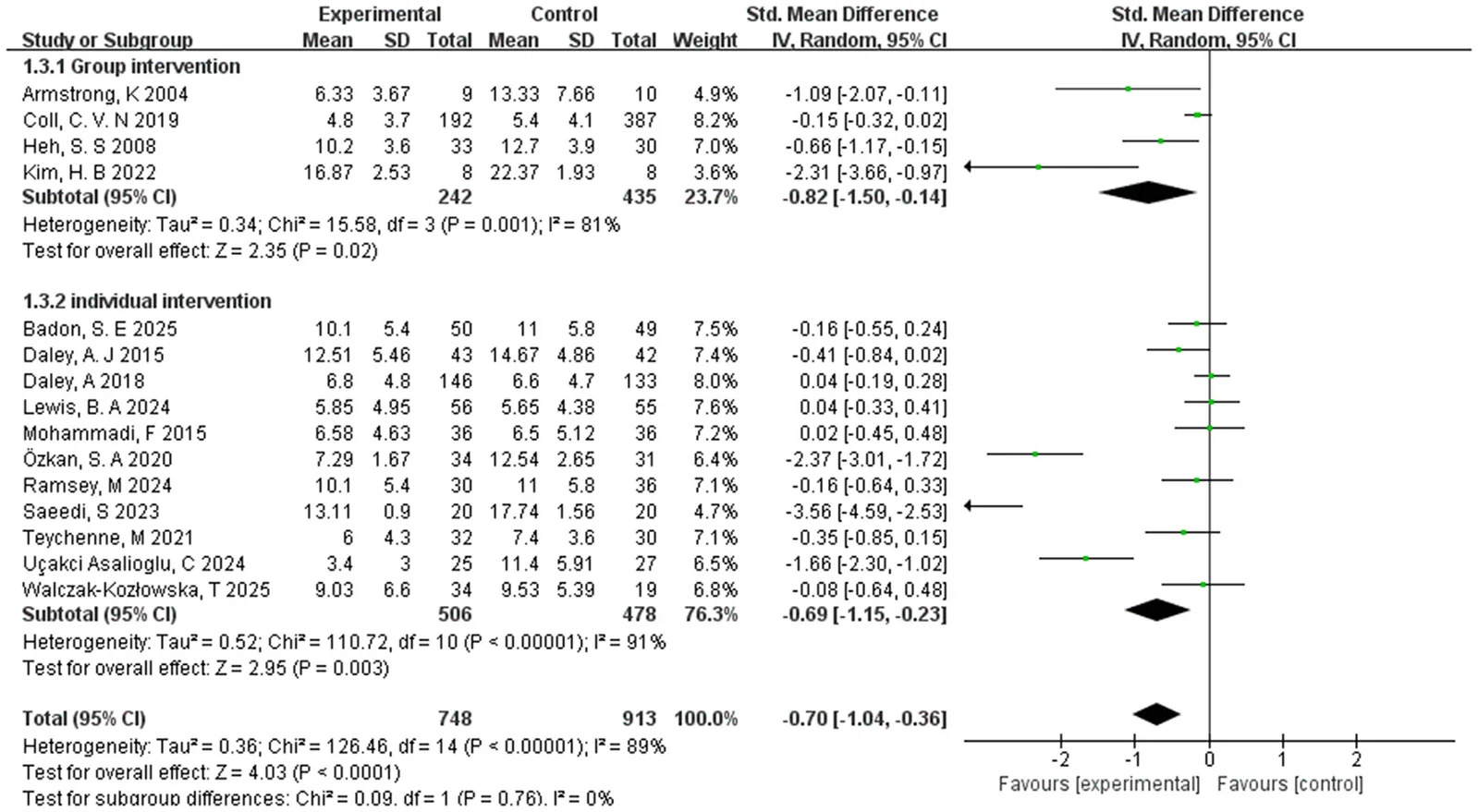
Forest plot of subgroup analyses by intervention format, suggesting that the two formats are statistically comparable.

Although the effect size for group-based interventions was numerically larger, the test for subgroup differences was not significant (χ^2^ = 0.09, df = 1, *p* = 0.76), as shown in Figure 6. Thus, both group and individual exercise programmes effectively alleviate postnatal depressive symptoms with similar efficacy. The observed tendency toward slightly greater effects in group formats may reflect additional benefits such as social support or enhanced motivation through interpersonal interaction; however, further research is required to substantiate these possibilities.

##### 3.4.4.2 Exercise modality

**Figure 7:**
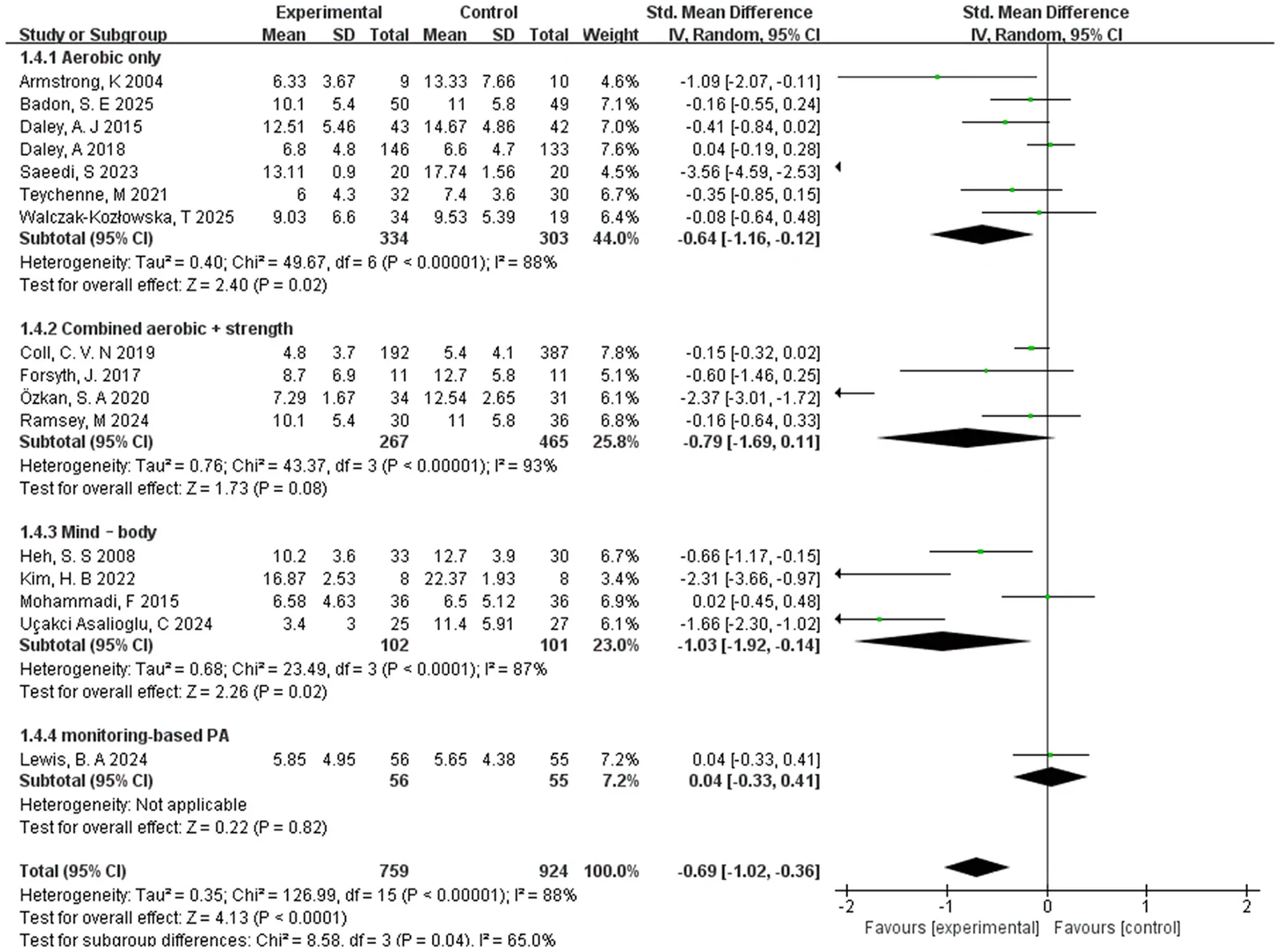
Forest plot of subgroup analyses by exercise modality.

When interventions were stratified by exercise modality, statistically significant differences in effect sizes were observed across categories (χ^2^ = 8.53, df = 3, *p* = 0.04). Mind–body interventions produced the largest and a statistically significant effect (SMD = −1.03, 95% CI: −1.92 to −0.14, *p* = 0.02), representing the strongest signal among all exercise types. Aerobic exercise alone also demonstrated a significant benefit (SMD = −0.64, 95% CI: −1.16 to −0.12, *p* = 0.02), supporting its efficacy in reducing postnatal depressive symptoms. Combined aerobic–strength training yielded a relatively large effect size (SMD = −0.79), but the confidence interval included zero (*p* = 0.08), indicating potential therapeutic value yet insufficient evidence for firm conclusions. By contrast, monitoring-based physical activity exhibited an effect size close to zero (SMD = −0.04, 95% CI: −0.33 to 0.41, *p* = 0.82), suggesting no observable benefit for postnatal depression (Figure 7).

Without inferring a hierarchical ranking of modalities, the overall pattern indicates that mind–body interventions provide the most consistent and robust evidence of benefit, aerobic exercise demonstrates reliable positive effects, and combined aerobic–strength programmes warrant further investigation. Interventions based solely on activity monitoring are not supported by current evidence for improving postnatal depressive symptoms.

##### 3.4.4.3 Timing of intervention initiation

**Figure 8:**
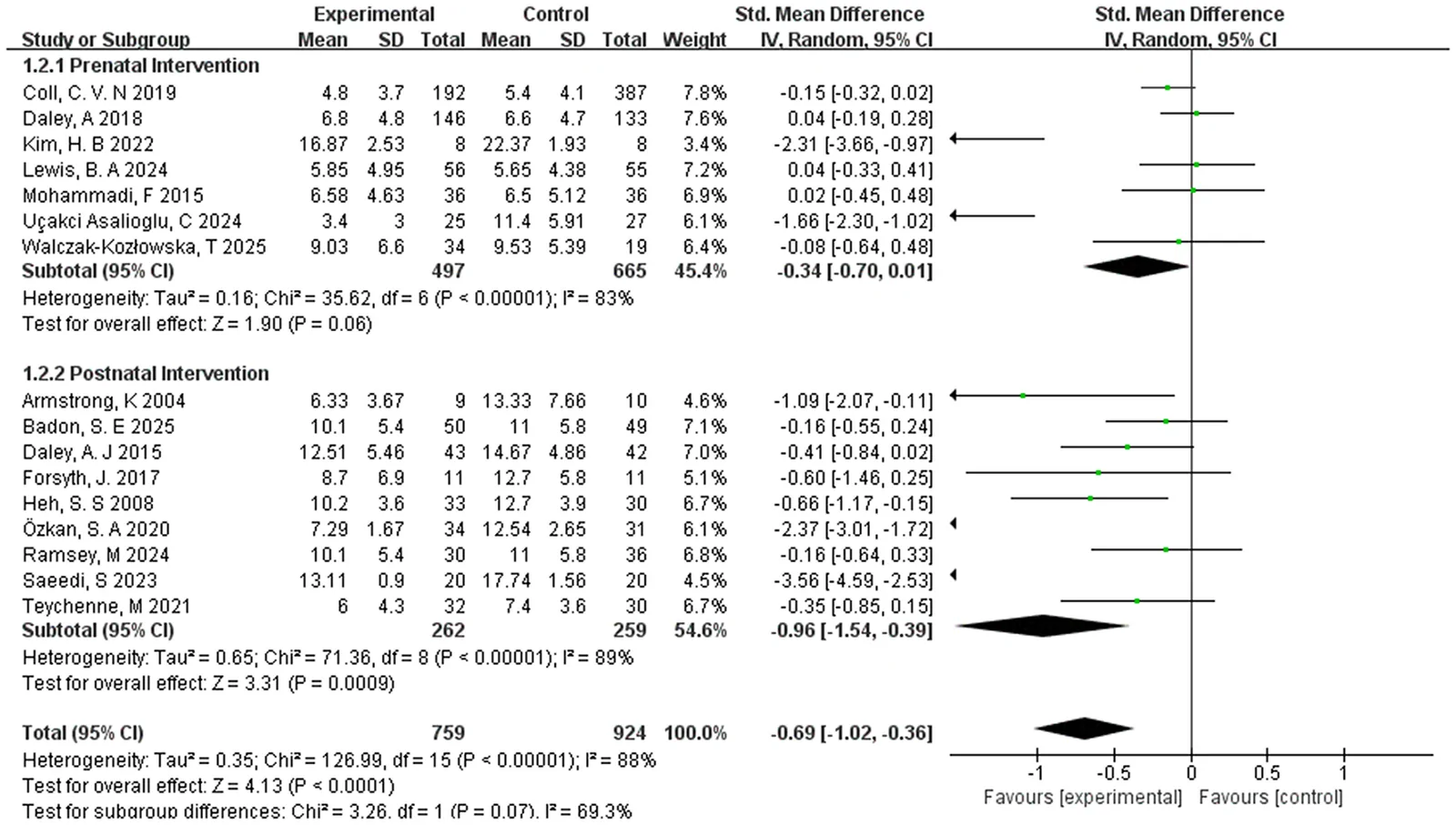
Forest plot of subgroup analyses by timing of intervention initiation.

The trials were grouped according to the timing of intervention initiation, distinguishing between prenatal and postnatal exercise programmes. Nine trials that introduced exercise during pregnancy (*n* = 497) yielded a pooled effect size of SMD = −0.34 (95% CI: −0.70 to 0.01, *P* = 0.06), indicating a tendency toward symptom improvement that did not reach statistical significance. By contrast, the seven trials initiating exercise in the postnatal period (*n* = 262) demonstrated a significant reduction in depressive symptoms, with a pooled effect of SMD = −0.96 (95% CI: −1.54 to −0.39, *P* = 0.0009) (Figure 8). These findings suggest that exercise interventions delivered after childbirth can exert a clear therapeutic effect.

Although the effect size for postnatal interventions was numerically larger, the subgroup difference test did not reach statistical significance (*P* > 0.05). Thus, the two initiation time points cannot be considered statistically different, and definitive claims of superiority are not supported. Nonetheless, the observed pattern suggests stronger improvement signals for postnatal initiation, a possibility that warrants further investigation in adequately powered trials.

Overall, exercise programmes initiated either during pregnancy or after birth appear to offer potential benefits for postnatal depression. Determining the optimal timing of intervention, however, will require additional high-quality research.

##### 3.4.4.4 Training frequency

The trials were categorized into three groups according to weekly training frequency: fewer than three sessions, three sessions, and more than three sessions per week. All three frequencies showed a general tendency toward improving postnatal depressive symptoms, although the magnitude of effects varied across groups.

**Figure 9:**
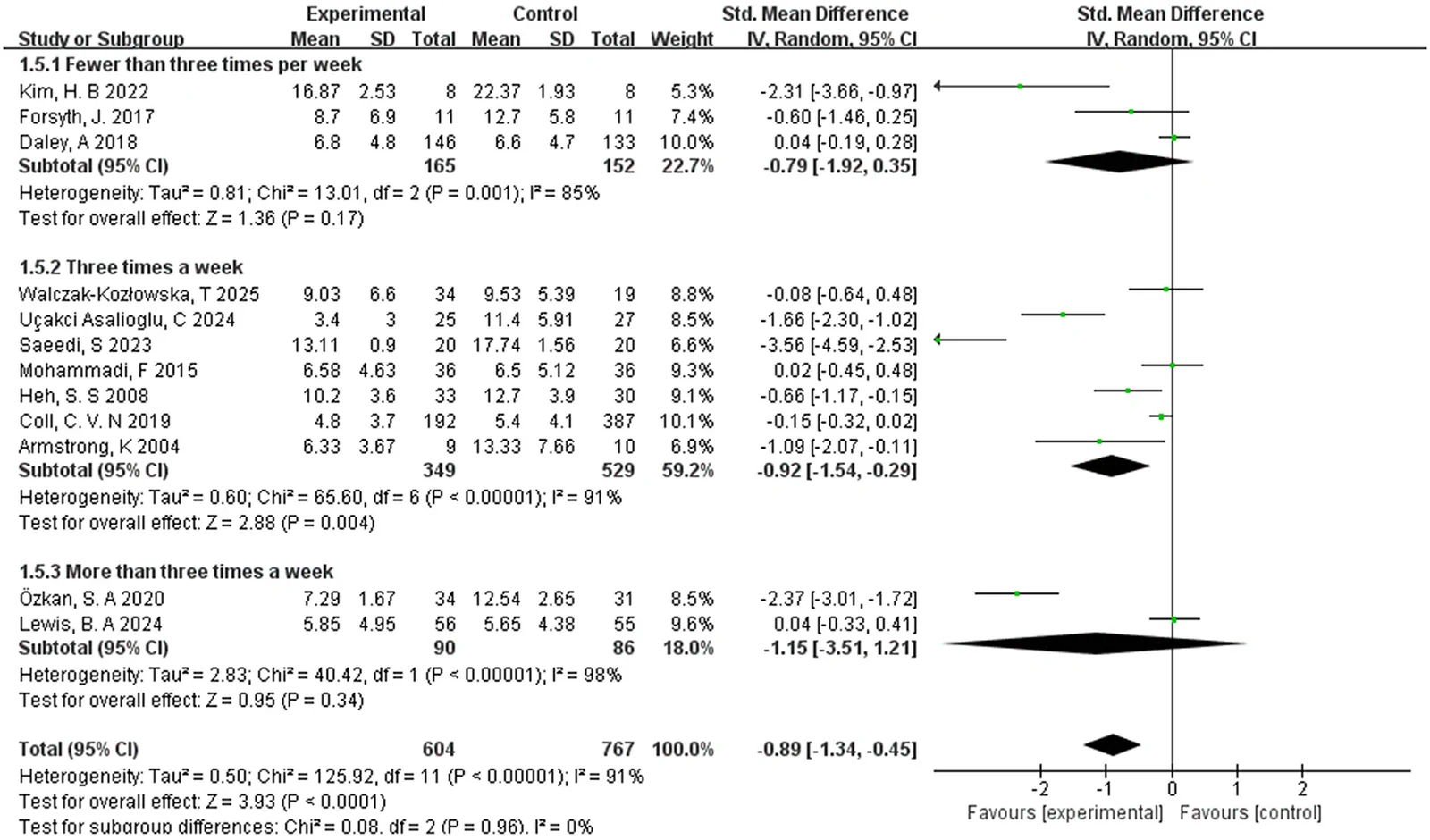
Forest plot of subgroup analyses by training frequency.

Interventions delivered fewer than three times per week produced a pooled effect size of SMD = −0.79 (95% CI: −1.92 to 0.35, *P* = 0.17), indicating a non-significant trend toward improvement. Trials prescribing three sessions per week demonstrated a significant reduction in depressive symptoms, yielding a pooled effect of SMD = −0.92 (95% CI: −1.54 to −0.29, P = 0.004), suggesting that this frequency may provide more consistent therapeutic benefits. Programmes involving more than 3 weekly sessions showed an improvement trend as well (SMD = −1.15), although the wide confidence interval (95% CI: −3.51 to 1.21, *P* = 0.34) crossing zero indicates insufficient evidence to support a definitive effect (Figure 9).

Despite numerical differences in effect magnitudes—most notably the consistent significance observed in the three-times-weekly subgroup—the formal test for subgroup differences did not reach statistical significance (*P* > 0.05). Accordingly, no frequency can be considered superior on statistical grounds. These findings collectively suggest that a range of training frequencies may yield benefits for postnatal depression, while the determination of an optimal exercise dose will require further well-powered randomized controlled trials.

##### 3.4.4.5 Intervention duration

**Figure 10:**
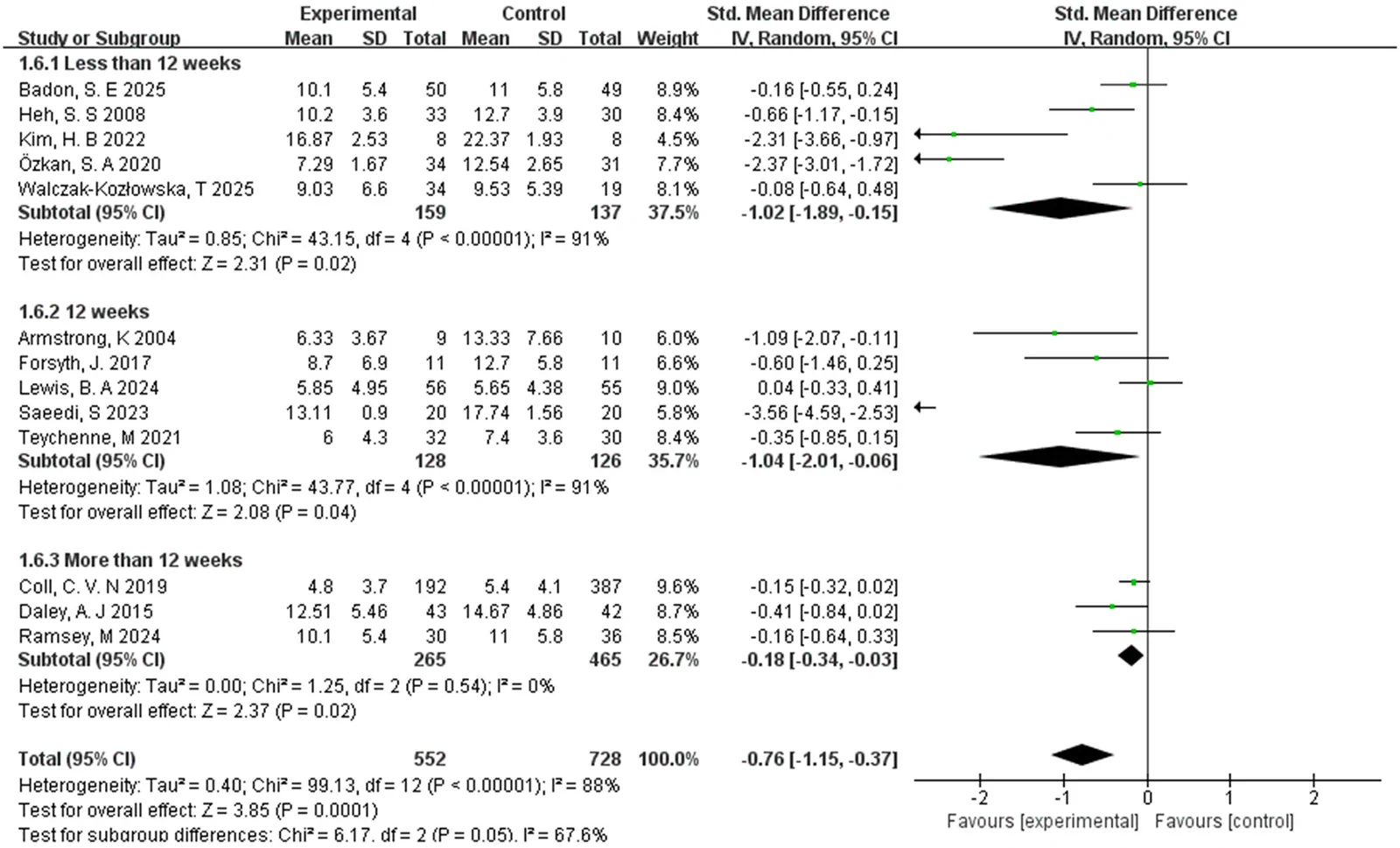
Forest plot of subgroup analyses by Intervention duration.

The trials were categorized into three groups according to the total duration of the exercise intervention: less than 12 weeks, 12 weeks, and more than 12 weeks. All three durations were associated with reductions in postnatal depressive symptoms, although the magnitude of effect varied substantially across groups. Interventions lasting fewer than 12 weeks demonstrated significant therapeutic benefit, with a pooled effect size of SMD = −1.02 (95% CI: −1.89 to −0.15, *P* = 0.02). Twelve-week interventions also produced statistically significant improvements, yielding a pooled effect of SMD = −1.04 (95% CI: −2.01 to −0.06, *P* = 0.04), comparable in magnitude to short-term programmes. By contrast, interventions extending beyond 12 weeks, while still statistically significant, showed a markedly attenuated effect (SMD = −0.18, 95% CI: −0.34 to −0.03, *P* = 0.02) (Figure 10).

The test for subgroup differences indicated a marginally significant distinction among the three duration categories (*P* = 0.05), suggesting that intervention length may influence the efficacy of exercise programmes for postnatal depression. Notably, the data exhibited a clear pattern in which therapeutic effects appeared to diminish with extended programme duration: the strongest effects were observed in interventions of fewer than 12 weeks, remained stable at 12 weeks, and declined substantially beyond this point. This attenuation could reflect reduced adherence over time, accumulated fatigue, or diminishing novelty of the intervention; however, such interpretations remain speculative given current evidence.

Overall, while interventions of all durations conferred measurable benefit, short- and medium-term programmes generated more pronounced improvement signals, whereas longer-term interventions may exhibit diminishing returns. Confirmation of this trend will require further well-designed randomized trials to refine understanding of the optimal duration for exercise-based interventions.

##### 3.4.4.6 Reporting of delivery mode

The included trials were classified into two categories based on whether the mode of delivery was reported: studies explicitly documenting vaginal delivery and those that did not specify delivery mode. Exercise interventions were associated with improvements in postnatal depressive symptoms across both categories, although the magnitude of benefit varied.

**Figure 11:**
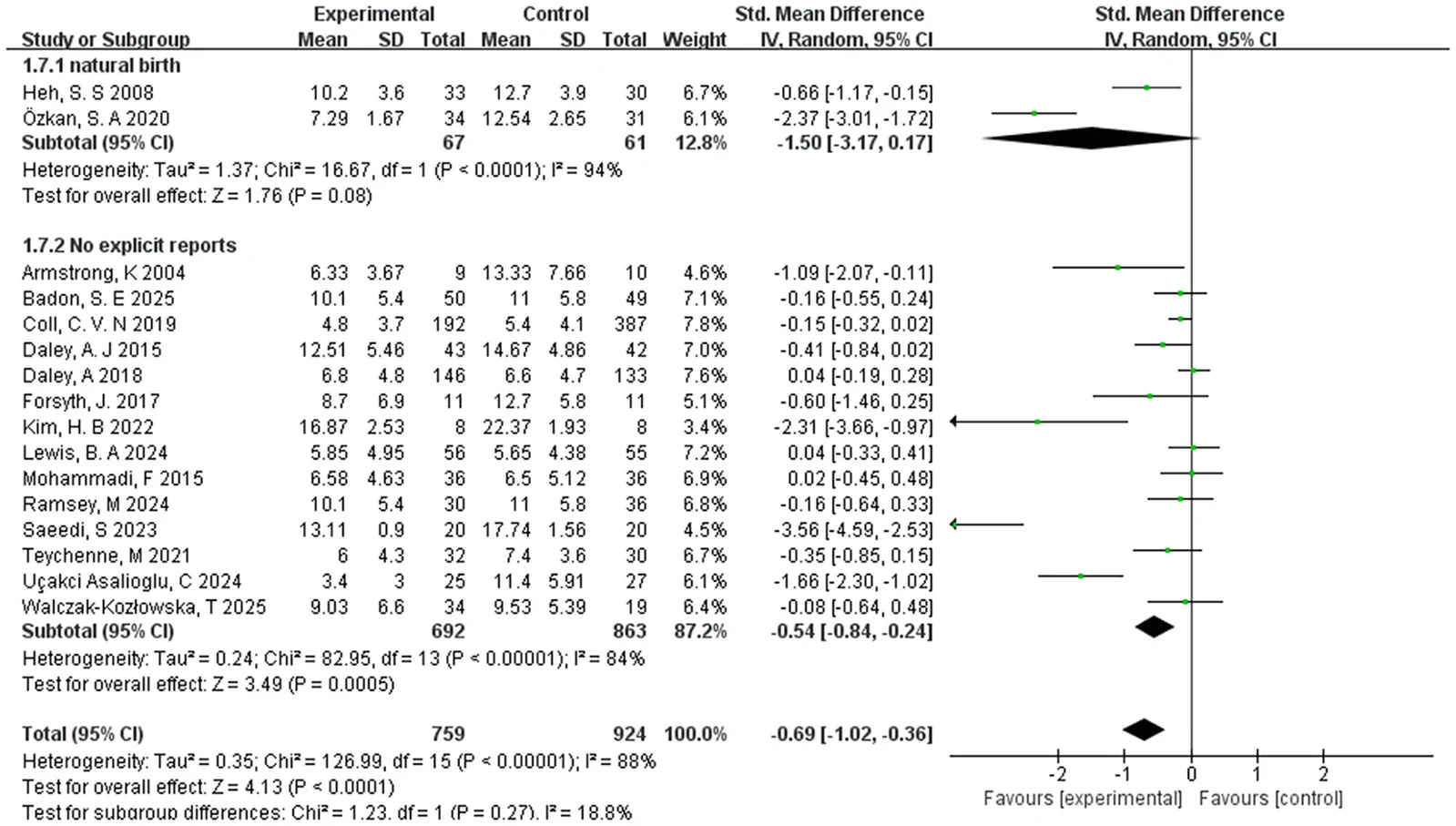
Forest plot of subgroup analyses by reporting of delivery mode.

Among studies reporting vaginal delivery, the pooled effect size was SMD = −1.50 (95% CI: −3.17 to 0.17, *P* = 0.08), indicating a clear trend toward symptom reduction that did not reach statistical significance. In contrast, studies without explicit reporting of delivery mode demonstrated a statistically significant effect, with a pooled SMD = −0.54 (95% CI: −0.84 to −0.24, *P* = 0.0005). Although the effect size was numerically larger in the vaginal-delivery group, the test for subgroup differences did not reach statistical significance (*P* > 0.05), indicating no statistically verifiable difference in efficacy between the two groups (Figure 11).

Nonetheless, the direction and magnitude of the pooled estimates suggest stronger improvement signals among women who delivered vaginally, raising the possibility that delivery mode may influence responsiveness to exercise interventions. This trend, while intriguing, remains preliminary and requires confirmation in future adequately powered trials. Overall, exercise interventions appear to confer psychological benefit irrespective of delivery-mode reporting. Future research would benefit from more consistent and detailed documentation of delivery-related variables to better assess their potential moderating role.

## 4 Discussion

This meta-analysis provides a comprehensive evaluation of the effects of exercise interventions on postnatal depression, offering important insights into the potential role of physical activity in supporting mental health during the postpartum period. Synthesizing evidence from 16 randomized controlled trials, the overall analysis demonstrated that exercise significantly alleviates depressive symptoms in postnatal women, with a pooled effect size indicating a moderate to large beneficial effect. These findings underscore the value of exercise as a viable non-pharmacological strategy for improving postnatal mental wellbeing.

To examine whether specific characteristics of exercise interventions influence their efficacy, six predefined subgroup analyses were conducted, encompassing intervention format, exercise modality, timing of intervention, training frequency, duration, and delivery mode reporting. Both individualized and group-based interventions produced comparable improvements in depressive symptoms. Although group-based programmes exhibited slightly larger effect sizes, the difference between the two formats did not reach statistical significance, indicating that both approaches constitute effective intervention options. Significant variation emerged across exercise modalities. Mind–body interventions produced the strongest and most consistent improvements, while pure aerobic exercise was also associated with significant benefit. Combined aerobic–strength training yielded relatively large effect sizes, but confidence intervals crossed zero, limiting the certainty of these findings. In contrast, monitoring-based physical activity showed no significant impact. These patterns suggest that exercise type may modulate therapeutic efficacy, highlighting mind–body interventions as a particularly promising avenue for future investigation.

With respect to the timing of intervention initiation, both prenatal and postnatal exercise programmes were associated with reductions in depressive symptoms. Although postnatal interventions showed numerically larger effects, the between-group comparison was not statistically significant. Thus, no definitive conclusion can be drawn regarding superiority. Nonetheless, the stronger improvement signals observed in postnatal programmes suggest the possibility of enhanced benefit, warranting confirmation in future trials. Across categories of training frequency, all three groups—fewer than three sessions per week, three sessions per week, and more than three sessions per week—demonstrated improvement trends, with the three-times-weekly programmes showing statistically significant effects. However, intergroup differences were not significant, precluding conclusions regarding an optimal frequency. These results suggest that multiple training frequencies may confer benefit, but clearer dose–response relationships remain to be established. Intervention duration revealed a marginally significant subgroup difference (*P* = 0.05). Short-term (<12 weeks) and medium-term (12 weeks) programmes demonstrated strong and significant effects, whereas those extending beyond 12 weeks showed markedly attenuated benefits. This apparent diminution over time may reflect declining adherence, psychological fatigue, or reduced novelty of the intervention, although current evidence is insufficient to support mechanistic conclusions. Finally, subgrouping based on delivery mode reporting revealed that studies explicitly documenting vaginal delivery did not achieve statistical significance but produced numerically larger effect sizes, whereas studies lacking explicit delivery-mode reporting showed significant effects. As the subgroup comparison was not statistically significant, no firm inferences can be drawn regarding differential responsiveness based on delivery mode. Nonetheless, the stronger improvement signals in the vaginal-delivery group raise the possibility of population-specific variation in treatment response.

In summary, this review demonstrates that exercise interventions consistently confer benefits for postnatal depression. Several intervention characteristics—particularly exercise modality and programme duration—may influence effect magnitude. While subgroup analyses identified a number of noteworthy trends, many between-group differences were not statistically significant, necessitating cautious interpretation. Further high-quality research is required to clarify optimal exercise dosage, the most effective intervention window, and individual-level moderators, thereby supporting the development of more precise and evidence-informed exercise strategies for postnatal mental health.

The findings of this study are broadly consistent with previous reviews demonstrating that exercise can significantly alleviate depressive symptoms in new mothers. A network meta-analysis by Wang et al. (2023) similarly reported moderate-to-large effects of exercise on postpartum depression; however, the quality of evidence was limited, and comparisons between exercise modalities were not robust (13). By applying a traditional random-effects model in combination with six predefined subgroup analyses, the present study not only corroborates the overall beneficial effect but also clarifies the more reliable efficacy of mind–body and aerobic exercises. Moreover, the lack of effect observed for monitoring-based activity provides more precise evidence regarding differential effectiveness across exercise types.

Savoia et al. (14), who investigated online interventions, reported substantially smaller effect sizes. The stronger effects observed in our analysis suggest that structured, in-person exercise programmes may confer greater therapeutic benefit, thereby offering a useful comparative framework for future research on digital mental-health interventions. Poyatos-León et al. (15) and McCurdy et al. (16) documented small-to-moderate effects of exercise but often analyzed interventions initiated during pregnancy and after childbirth together, without clear distinction by intervention onset. Furthermore, these earlier reviews did not systematically examine key components of the “exercise prescription,” such as exercise modality, training frequency, or programme duration. Although some of the trials included in the present study commenced during pregnancy, all outcomes focused explicitly on postnatal depressive symptoms, thereby avoiding the interpretive limitations associated with mixed perinatal analyses. Importantly, this study expands prior work by systematically evaluating the potential influence of intervention timing, frequency, duration, and exercise modality through comprehensive subgroup analyses, addressing a critical evidence gap concerning how exercise interventions should be optimally implemented. While Pentland et al. (17) focused specifically on walking interventions, our results show that although walking is beneficial, mind–body exercise modalities exhibit stronger improvement signals, thus broadening the range of exercise strategies with demonstrated potential.

Together, these findings make two key contributions: first, they confirm the significant therapeutic value of exercise for postnatal depression; and second, they provide more granular and methodologically refined evidence—particularly regarding the characteristics of exercise interventions—than has been available in the existing literature. This enriched evidence base offers an important foundation for developing more targeted and effective exercise prescriptions in future clinical and public-health practice.

The antidepressant effects of exercise in the postnatal period likely arise from the interplay of multiple biological and psychosocial mechanisms (18). Biologically, exercise has been shown to increase the secretion of brain-derived neurotrophic factor (BDNF) and endorphins, thereby enhancing neural plasticity and supporting more effective emotional regulation—mechanisms that are well established in the broader depression literature (19, 20). Exercise also modulates hypothalamic–pituitary–adrenal (HPA) axis activity, attenuating stress-hormone responses and reducing the heightened physiological stress often observed after childbirth (21).

From a psychosocial perspective, regular exercise offers a range of benefits for new mothers. It can enhance self-efficacy, improve sleep quality, alleviate postnatal fatigue and somatic discomfort, and provide a structured opportunity for self-care (22, 23). Group-based and mind–body exercise formats may additionally confer social support, emotional resonance, and relaxation effects, which collectively contribute to mood improvement (24). Exercise can also help mothers re-establish daily routines and regain a sense of personal identity—factors that are particularly relevant during the substantial role transitions that characterize the postpartum period (25, 26). Taken together, these biological, psychological, and social pathways provide a coherent theoretical framework for understanding the beneficial effects of exercise on postnatal depression. Further research is warranted to determine whether different exercise modalities exert their effects through distinct mechanisms, which may ultimately inform the development of more targeted and personalized exercise prescriptions.

This study offers several notable strengths. By restricting inclusion to randomized controlled trials, it ensures a high level of evidentiary rigor. In addition to quantifying the overall effect of exercise on postnatal depression, the study systematically examined key components of exercise prescription—including modality, frequency, intervention timing, and duration—through six structured subgroup analyses. This approach provides a more granular understanding of how specific intervention characteristics may influence outcomes. The use of standardized effect sizes also enabled the integration of findings across diverse depression assessment tools, thereby enhancing comparability.

Nonetheless, several limitations should be acknowledged. First, some subgroup analyses were limited by relatively small numbers of included studies and participants, resulting in wide confidence intervals and potentially unstable effect estimates. Second, the methodological quality of the included trials may have influenced the interpretation of the pooled effects. Due to the nature of exercise interventions, blinding of participants and personnel was often difficult to achieve, which may have introduced performance and detection bias, particularly when depressive symptoms were assessed using self-reported measures. In addition, unclear allocation concealment in some studies may have increased the risk of selection bias, while small sample sizes may have contributed to imprecise or potentially exaggerated effect estimates. Furthermore, residual heterogeneity may be attributable to variations in exercise intensity, intervention structure, participant characteristics, and cultural contexts. Finally, inconsistent reporting of intervention adherence limited our ability to evaluate dose–response relationships and identify optimal exercise characteristics.

Despite these limitations, the findings reinforce that exercise represents a feasible, accessible, and effective non-pharmacological option for managing postnatal depression. Clinical and public-health practice may benefit from integrating structured exercise programmes into routine postpartum care, with particular emphasis on low-cost formats such as aerobic and mind–body exercises. Facilitating group-based or supervised sessions may further enhance adherence and motivational engagement. Future work should prioritize larger, well-designed trials with clearer reporting of intervention adherence and exercise parameters to refine evidence-based exercise prescriptions for this population.

## 5 Conclusion

This systematic meta-analysis demonstrates that exercise interventions confer significant reductions in postnatal depressive symptoms, supporting their role as a safe, accessible and effective non-pharmacological treatment option. The overall effect sizes indicate moderate to substantial clinical benefits. Subgroup analyses further suggest that intervention characteristics—particularly exercise modality and duration—may influence efficacy. Mind–body exercises yielded the most pronounced and consistent improvements, while programmes of 12 weeks or shorter appeared to produce more robust therapeutic effects than longer interventions.

Although several subgroup differences did not reach statistical significance and should therefore be interpreted cautiously, the present findings provide an important evidence base for understanding how specific elements of exercise prescriptions may shape treatment outcomes. Taken together, the results highlight the clinical and public-health value of exercise as a low-risk, potentially scalable approach for supporting mental health in the postnatal period. Future research should prioritize the optimisation of exercise dosage, timing and programme structure, alongside improved reporting of intervention fidelity and participant adherence. Such efforts will be essential for refining targeted, evidence-based exercise strategies that can be effectively integrated into routine postnatal care.

## Data Availability

The original contributions presented in the study are included in the article/Supplementary material, further inquiries can be directed to the corresponding author.
