# Supplementary material for: Which exercise strategies improve postpartum depression? A systematic review and meta-analysis of randomized controlled trials
Source: Front Public Health. 2026 Aug 27;14:1876038. doi: 10.3389/fpubh.2026.1876038 (PMC13557993; doi:10.3389/fpubh.2026.1876038)
Supplement: Supplementary file 1 [file Table_2.pdf]

	Random sequence generation (selection bias)	Allocation concealment (selection bias)	Blinding of participants and personnel (performance bias)	Blinding of outcome assessment (detection bias)	Incomplete outcome data (attrition bias)	Selective reporting (reporting bias)	Other bias
Armstrong, K 2004	+	+	−	?	+	+	?
Badon, S. E 2025	+	+	−	?	+	+	?
Coll, C. V. N 2019	+	?	−	−	+	+	?
Daley, A. J 2015	+	+	−	?	+	+	?
Daley, A 2018	+	+	−	?	+	+	?
Forsyth, J. 2017	+	+	−	+	+	+	+
Heh, S. S 2008	?	?	−	+	+	+	?
Kim, H. B 2022	?	?	−	−	+	?	+
Lewis, B. A 2024	+	+	−	+	+	?	?
Mohammadi, F 2015	+	+	−	−	?	+	?
Özkan, S. A 2020	+	?	−	+	+	+	+
Ramsey, M 2024	+	+	−	+	+	+	?
Saeedi, S 2023	?	?	−	?	+	+	?
Teychenne, M 2021	+	+	−	+	+	?	+
Uçakci Asalioglu, C 2024	+	+	−	−	+	+	+
Walczak-Kozłowska, T 2025	+	+	−	?	+	+	+
