# Supplementary material for: Which exercise strategies improve postpartum depression? A systematic review and meta-analysis of randomized controlled trials
Source: Front Public Health. 2026 Aug 27;14:1876038. doi: 10.3389/fpubh.2026.1876038 (PMC13557993; doi:10.3389/fpubh.2026.1876038)
Supplement: Supplementary file 2 [file Table_1.docx]

**Pubmed search algorithm**

#1	"Depression"[Mesh Terms]

#2	"depressive symptoms"[Title/Abstract] OR "depressive symptom"[Title/Abstract] OR "symptom depressive"[Title/Abstract] OR "emotional depression"[Title/Abstract] OR "depression emotional"[Title/Abstract]

#3	#1 OR #2

#4	"Exercise"[Mesh Terms]

#5 "Exercises"[Title/Abstract] OR "physical activity"[Title/Abstract] OR "activities physical"[Title/Abstract] OR "activity physical"[Title/Abstract] OR "physical activities"[Title/Abstract] OR "exercise physical"[Title/Abstract] OR "exercises physical"[Title/Abstract] OR "physical exercise"[Title/Abstract] OR "physical exercises"[Title/Abstract] OR "acute exercise"[Title/Abstract] OR "acute exercises"[Title/Abstract] OR "exercise acute"[Title/Abstract] OR "exercises acute"[Title/Abstract] OR "exercise isometric"[Title/Abstract] OR "exercises isometric"[Title/Abstract] OR "isometric exercises"[Title/Abstract] OR "isometric exercise"[Title/Abstract] OR "exercise aerobic"[Title/Abstract] OR "aerobic exercise"[Title/Abstract] OR "aerobic exercises"[Title/Abstract] OR "exercises aerobic"[Title/Abstract] OR "exercise training"[Title/Abstract] OR "exercise trainings"[Title/Abstract] OR "training exercise"[Title/Abstract] OR (("education"[MeSH Subheading] OR "education"[All Fields] OR "Training"[All Fields] OR "education"[MeSH Terms] OR "train"[All Fields] OR "train s"[All Fields] OR "trained"[All Fields] OR "training s"[All Fields] OR "Trainings"[All Fields] OR "trains"[All Fields]) AND "Exercise"[Title/Abstract])

#6	#4 OR #5

#7 "Postpartum Period "[Mesh Terms]

#8 "Period, Postpartum"[Title/Abstract] OR "Postpartum"[Title/Abstract] OR "Postpartum Women"[Title/Abstract] OR "Women, Postpartum"[Title/Abstract] OR "Puerperium"[Title/Abstract]

#9	#7 OR #8

#10	"randomized controlled trial"[Publication Type] OR "randomized"[Title/Abstract] OR "placebo"[Title/Abstract]

#11	#3 AND #6 AND #9 AND #10

**Web of Science search algorithm**

#1 TS=(“Depression” OR “Depressive Symptoms” OR “Depressive Symptom” OR “Symptom, Depressive” OR “Emotional Depression” OR “Depression, Emotional”) and Preprint Citation Index (Exclude – Database)

#2 TS=(“Exercise, Acute” OR “Exercises, Acute” OR “Exercise, Isometric” OR “Exercises, Isometric” OR “Isometric Exercises” OR “Isometric Exercise” OR “Exercise, Aerobic” OR “Aerobic Exercise” OR “Aerobic Exercises” OR “Exercises, Aerobic” OR “Exercise Training” OR “Exercise Trainings” OR “Training, Exercise” OR “Trainings, Exercise”) and Preprint Citation Index (Exclude – Database)

#3 TS=(“Postpartum Period” OR “Period, Postpartum” OR “Postpartum” OR “Postpartum Women” OR “Women, Postpartum” OR “Puerperium”) and Preprint Citation Index (Exclude – Database)

#4 TS=(“randomized controlled trial” OR “randomized” OR “placebo” OR “RCT”) and Preprint Citation Index (Exclude – Database)

#5 #1 AND #2 AND #3 AND #4 and Preprint Citation Index (Exclude – Database)

**Embase search algorithm**

#1	'depression'/exp OR depression

#2	'depressive symptoms':ab,ti OR 'depressive symptom':ab,ti OR 'symptom, depressive':ab,ti OR 'emotional depression':ab,ti OR 'depression, emotional':ab,ti

#3	#1 OR #2

#4	exercise

#5	'exercises':ab,ti OR 'physical activity':ab,ti OR 'activities, physical':ab,ti OR 'activity, physical':ab,ti OR 'physical activities':ab,ti OR 'exercise, physical':ab,ti OR 'exercises, physical':ab,ti OR 'physical exercise':ab,ti OR 'physical exercises':ab,ti OR 'acute exercise':ab,ti OR 'acute exercises':ab,ti OR 'exercise, acute':ab,ti OR 'exercises, acute':ab,ti OR 'exercise, isometric':ab,ti OR 'exercises, isometric':ab,ti OR 'isometric exercises':ab,ti OR 'isometric exercise':ab,ti OR 'exercise, aerobic':ab,ti OR 'aerobic exercise':ab,ti OR 'aerobic exercises':ab,ti OR 'exercises, aerobic':ab,ti OR 'exercise training':ab,ti OR 'exercise trainings':ab,ti OR 'training, exercise':ab,ti OR 'trainings, exercise':ab,ti

#6	#4 OR #5

#7	'Postpartum Period':ab,ti OR 'Period, Postpartum':ab,ti OR 'Postpartum':ab,ti OR 'Postpartum Women':ab,ti OR 'Women, Postpartum':ab,ti OR 'Puerperium':ab,ti

#8	'randomized controlled trial':ab,ti OR 'randomized':ab,ti OR 'placebo':ab,ti OR 'rct':ab,ti

#9	#3 AND #6 AND #7 AND #8

**Cochrane Library search algorithm**

#1	Depression

#2	(Depressive Symptoms):ab,ti,kw OR (Depressive Symptom):ab,ti,kw OR (Symptom, Depressive):ab,ti,kw OR (Emotional Depression):ab,ti,kw OR (Depression, Emotional):ab,ti,kw

#3	#1 OR #2

#4	exercise

#5	(Exercises):ab,ti,kw OR (Physical Activity):ab,ti,kw OR (Activities, Physical):ab,ti,kw OR (Activity, Physical):ab,ti,kw OR (Physical Activities):ab,ti,kw OR (Exercise, Physical):ab,ti,kw OR (Exercises, Physical):ab,ti,kw OR (Physical Exercise):ab,ti,kw OR (Physical Exercises):ab,ti,kw OR (Acute Exercise):ab,ti,kw OR (Acute Exercises):ab,ti,kw OR (Exercise, Acute):ab,ti,kw OR (Exercises, Acute):ab,ti,kw OR (Exercise, Isometric):ab,ti,kw OR (Exercises, Isometric):ab,ti,kw OR (Isometric Exercises):ab,ti,kw OR (Isometric Exercise):ab,ti,kw OR (Exercise, Aerobic):ab,ti,kw OR (Aerobic Exercise):ab,ti,kw OR (Aerobic Exercises):ab,ti,kw

#6	#4 OR #5

#7	(Postpartum Period):ab,ti,kw OR (Period, Postpartum):ab,ti,kw OR (Postpartum):ab,ti,kw OR (Postpartum Women):ab,ti,kw OR (v):ab,ti,kw OR (Depression, Emotional):ab,ti,kw OR (Puerperium):ab,ti,kw

#8 (randomized controlled trial):ab,ti,kw OR (randomized):ab,ti,kw OR (placebo):ab,ti,kw OR (RCT):ab,ti,kw

#9	#3 AND #6 AND #7 AND #8
